# Specific T-cell responses for guiding treatment with convalescent plasma in severe COVID-19 and humoral immunodeficiency: a case report

**DOI:** 10.1186/s12879-022-07323-4

**Published:** 2022-04-11

**Authors:** Katarina Nyström, Maria Hjorth, Ramona Fust, Åsa Nilsdotter-Augustinsson, Marie Larsson, Katarina Niward, Sofia Nyström

**Affiliations:** 1 Department of Infectious Diseases, and Department of Biomedical and Clinical Sciences, Linköping University, Linköping, Sweden; 2 Department of Clinical Immunology and Transfusion Medicine and Department of Biomedical and Clinical Sciences, Linköping University, Linköping, Sweden; 3 Division of Molecular Medicine and Virology, Department of Biomedical and Clinical Sciences, Linköping University, Linköping, Sweden

**Keywords:** Case report, COVID-19, XLA, T-cell response, Convalescent plasma therapy

## Abstract

**Background:**

The immune response to SARS-CoV-2 virus, the cause of COVID-19, is complex. Antibody mediated responses are important for viral clearance but may also drive hyperinflammation in severe COVID-19. We present a case of an individual with a genetic inability to produce antibodies and severe COVID-19, receiving no other specific anti-viral treatment than convalescent COVID-19 plasma, illustrating that hyperinflammation can occur in the absence of a humoral anti-viral response. In addition, the case illustrates that the assessment of SARS-CoV-2 T cell responses can facilitate clinical decision making in patients with COVID-19 and weak or absent humoral immune responses.

**Case presentation:**

A male with X-linked agammaglobulinemia on regular immunoglobulin replacement therapy, hospitalized for 35 days due to severe COVID-19. Systemic inflammatory parameters were highly elevated. After treatment with convalescent COVID-19 plasma he became afebrile and the fatigue diminished. He was discharged on day 42 and nasopharyngeal SARS-CoV-2 PCR eventually was negative on day 49. Evidence of SARS-CoV-2 specific T cells prior to administration of plasma therapy suggested that antibodies were crucial for viral clearance. Regular assessment showed robust and persistent SARS-CoV-2 specific T-cell responses after recovery suggested that prophylactic administration of convalescent COVID-19 plasma was unnecessary.

**Conclusion:**

Assessment of SARS-CoV-2T-cell responses can facilitate the clinical management of COVID-19 patients with humoral immunodeficiencies.

**Supplementary Information:**

The online version contains supplementary material available at 10.1186/s12879-022-07323-4.

## Background

Individuals with primary antibody deficiencies, such as common variable immune deficiency (CVID) and X-linked agammaglobulinemia (XLA) will depend on their T-cell responses for viral clearance of emerging infections such as COVID-19, since available preparations used for immunoglobulin (Ig) replacement therapy were collected prior to the COVID-19 pandemic. A mild clinical course of COVID-19 in individuals with CVID and XLA suggested that antibodies are superfluous in the clearance of the infection [1], which is supported by the observation of virologic control in the absence of a detectable B cell responses after B-cell depletion therapy [2]. Functional, potentially protective T-cell responses have been reported, also in the absence of detectable humoral responses in mild or asymptomatic COVID-19 [3]. On the other hand, hospitalized XLA-patients improved promptly after receiving COVID-19 convalescent (CC) -plasma [4, 5] and, CC-plasma therapy has also proved beneficial in patients subjected to B-cell depletion therapy [6]. We present a case with XLA and protracted, severe COVID-19 pneumonia and systemic hyperinflammation. He required high flow oxygen therapy (HFNO) and was not subjected to any COVID-19 specific therapy before treatment with CC-plasma. He recovered promptly after receiving CC-plasma. SARS-CoV-2 specific T-cells were detected before administration of CC-plasma suggesting that SARS-CoV-2 antibodies are important for viral clearance in severe COVID-19 in addition to virus specific T-cells. Regular assessments showed a durable and robust SARS-CoV-2 specific T-cell response that may protect from re-infection of SARS-CoV-2.

## Case presentation

**Fig. 1:**
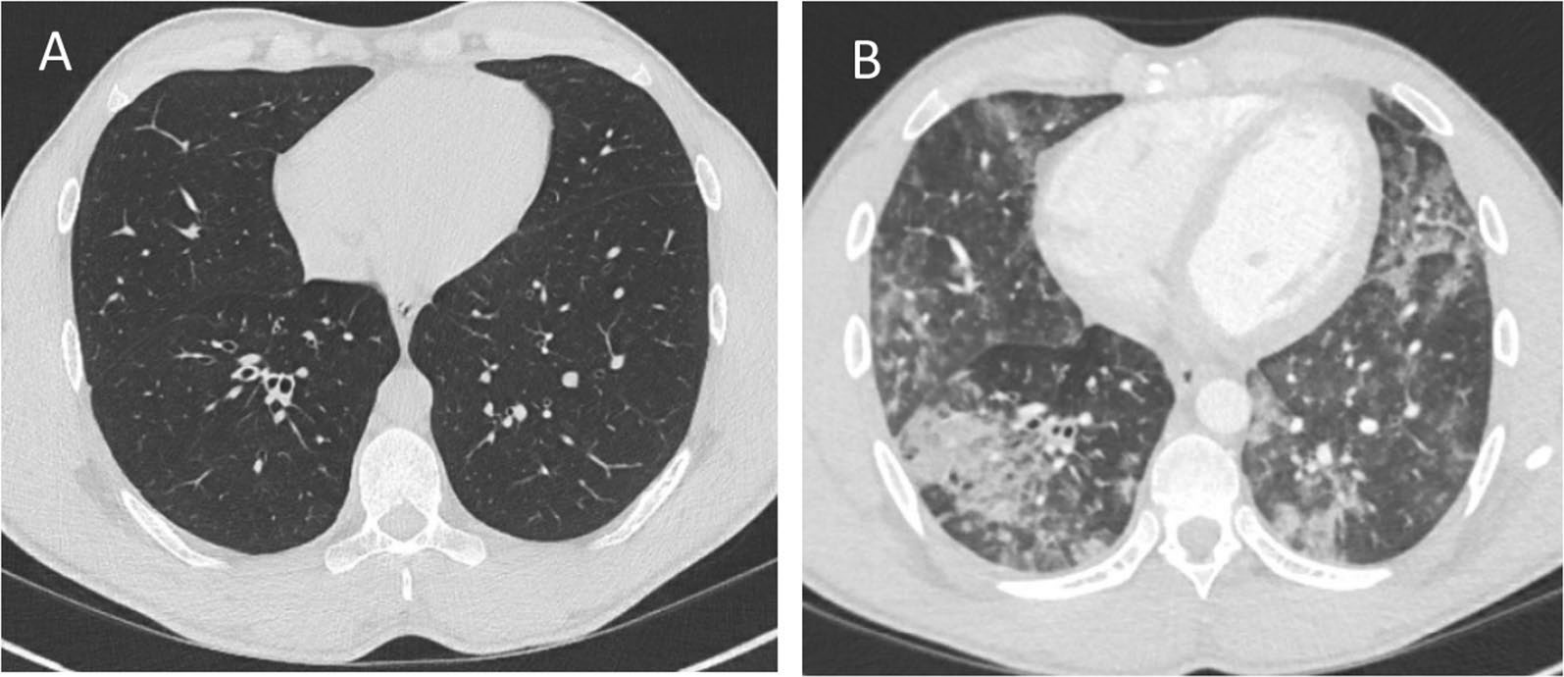
Chest images of patient before (**A**) and during COVID-19 showing diffuse bilateral ground-glass opacities (**B**)

**Fig. 2:**
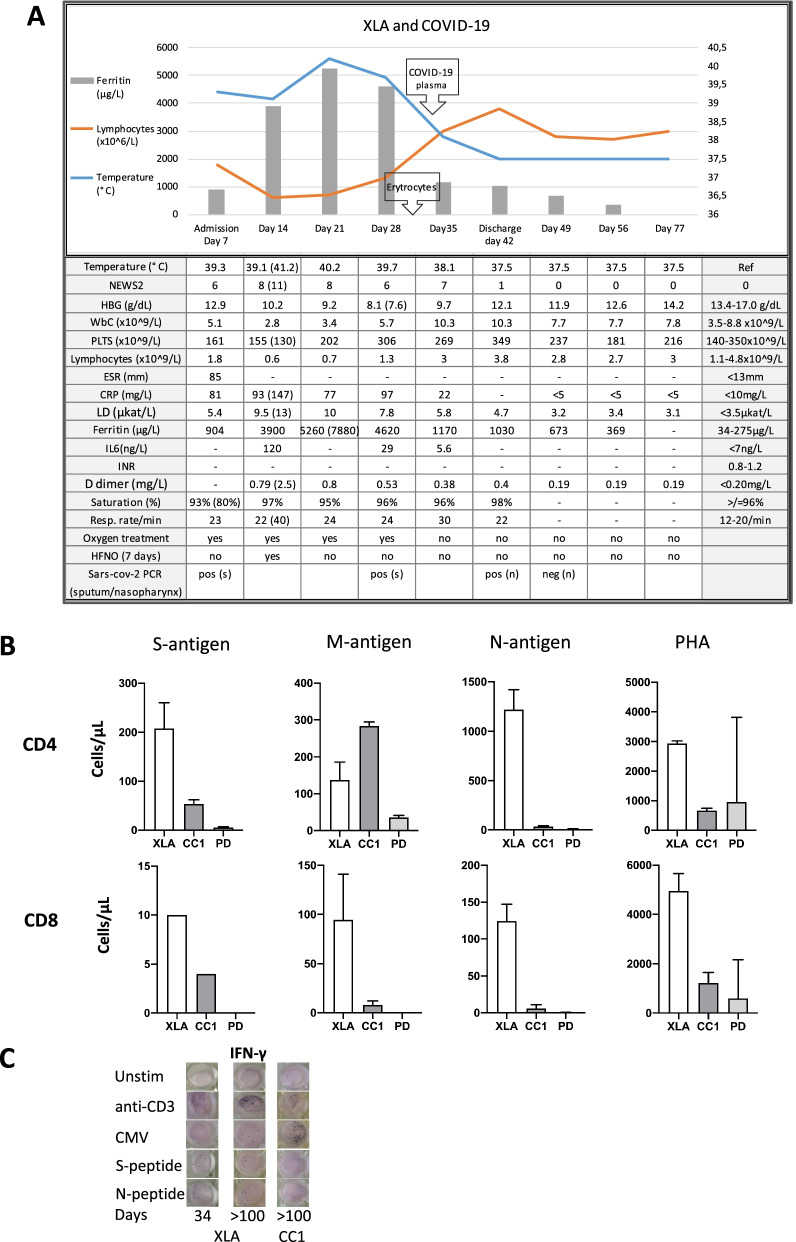
Summary of clinical and laboratory parameters during hospitalization and SARS-CoV-2 specific T cell responses. **A** Trajectory of clinical parameters during and post hospitalization of the patient. The arrows indicate time of transfusion of red cell concentrates and convalescent plasma therapy. Values in parentheses show the highest/lowest value during the period of hospitalization. NEWS2, National Early Warning Score; HFNO, High Flow Nasal Oxygen. **B** Quantification of proliferating T-cells in whole blood stimulated with specific SARS-CoV-2 peptides [spike (S), membrane (M) and nucleocapsid (N)] and the mitogen phytohemagglutinin (PHA). Medians and range of 2 experiments in XLA patient, convalescent control (CC), and 5 plasma donors (PD) are shown. **C** ELISpot showing IFN-γ-producing cells after stimulation with anti-CD3, CMV peptides and SARS-CoV-2 spike (S) and nucleocapsid (N) peptides

**Fig. 3:**
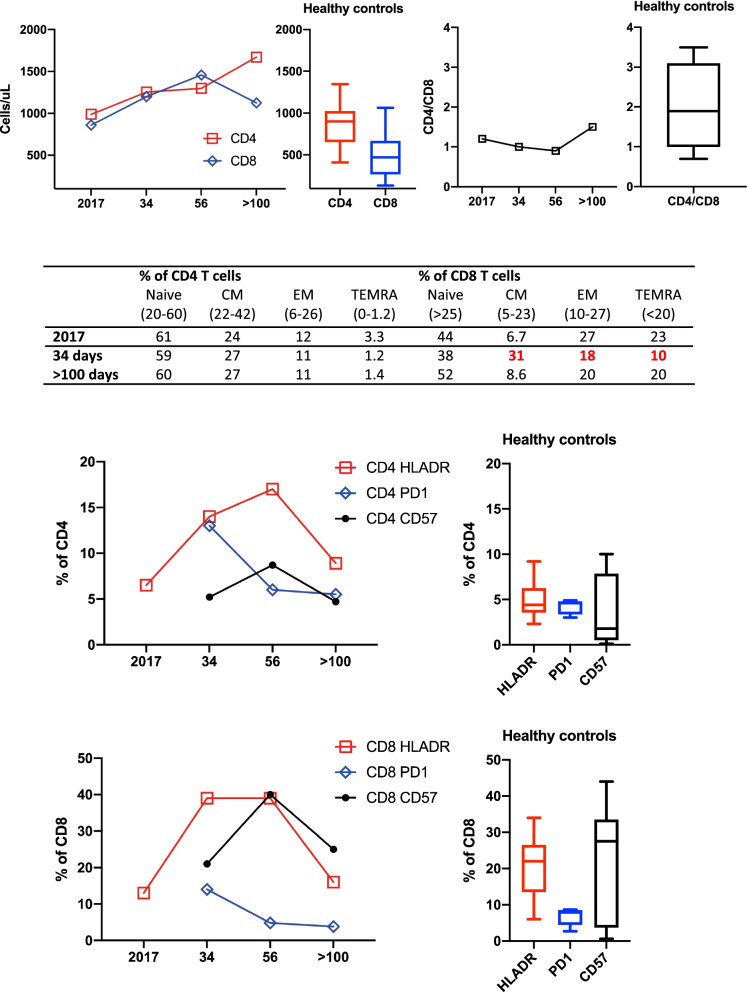
Extended T-cell phenotyping of peripheral blood by multi-color flow cytometry, before (2017), during (day 34) and after recovery (day 56 and day > 100) from COVID-19. Median and range of 10–11 healthy controls are shown for comparison

We report a 30-year-old male with XLA who was hospitalized for 35 days due to severe COVID-19 during the first wave of the pandemic; he received convalescent plasma and developed a strong SARS-CoV-2 specific T-cell response. The patient has been confirmed to have a hemizygote mutation, c. [763C > T], of nonsense in the gene encoding *Bruton’s tyrosine kinase* (BTK), resulting in the absence of mature B-cells and panhypogammaglobulinemia. The patient is on regular Ig-replacement therapy (170 mg/kg/week) and has minor bronchiectasis in the lower right lobe, normal spirometry and diffusion capacity. Upon admission he presented with one-week history of high fever, dry cough, and dyspnea. Treatment with oxygen, paracetamol, cefotaxime, doxycycline and low molecular heparin was immediately initiated. Positive SARS-CoV-2 PCR in sputum confirmed the diagnosis of COVID-19 at time of admission. Moreover, *S. pneumonia* and *H. influenza* were isolated in sputum culture. Chest computed tomography showed extensive bilateral infiltrates of ground-glass appearance and even consolidations (Fig. 1). Fever persisted and his condition deteriorated during day 14–21 with severe fatigue, highly elevated plasma levels of CRP, interleukin-6 and ferritin, and decreased lymphocyte counts (Fig. 2A). HFNO was given day 12–19 to maintain oxygen saturation above 94%. The patient did not receive any corticosteroids or antiviral therapy, since it was not part of the treatment regimen in Sweden at that time. Extended immune phenotyping of peripheral lymphocytes at day 34, showed an increase in the frequencies of helper T-cells and cytotoxic T-cells expressing the activation marker HLA-DR and the checkpoint molecule PD-1, and a shift towards central memory cells within the cytotoxic T-cell compartment (Fig. 3). We hypothesized that SARS-CoV-2 specific antibodies may be vital for viral clearance and to mitigate the inflammatory response. Plasma transfusions are considered safe, and SARS-CoV-2 spike protein neutralizing monoclonal antibodies (mAbs) were not available, hence CC-plasma therapy was initiated at day 35. The patient was administered 300 mL AB0-matched CC-plasma on two consecutive days without any transfusion-related adverse reactions. At this time, the fever had already started to decline, but after the plasma transfusions the patient became afebrile for the first time in 40 days (Fig. 2A). The fatigue diminished and he was mobilized and discharged on day 42. Nasopharyngeal sample was still PCR-positive for SARS-CoV-2 at this time point, but negative in a sample collected at day 49 (Fig. 2A).

SARS-CoV-2 specific T-cells was detected by a flow-cytometric assay for specific cell-mediated immune-response in activated whole blood [7]. Detailed information about cellular assays and immune phenotyping is available in Additional file 1: Supplementary Methods. Higher numbers of CD4 and CD8 T-cells, specific for SARS-CoV-2 spike- (S) or nucleocapsid (N) protein peptide-pools, were detected in peripheral blood of the patient compared to a seropositive individual that had tested PCR positive for SARS-CoV-2 with moderate COVID-19 and seropositive CC-plasma donors with mild COVID-19 (Fig. 2B). High frequencies of circulating SARS-CoV-2 specific CD4 and CD8 T-cells were detected in peripheral blood of the patient more than 200 days after recovery of COVID-19 (results not shown). In addition, functional SARS-CoV-2-specific memory T-cell responses were assessed in peripheral blood lymphocytes collected at day 34, prior to plasma transfusion, and at day 113 after disease onset. Interferon-γ producing cells were detected after stimulation with S- and N-peptides at both time-points (Fig. 2C). Only sporadic cytomegaly virus (CMV)-specific cells were detected at day 34, however after recovery of COVID-19 the number of CMV-specific cells were increased (Fig. 2C). After discharge, the patient received prophylactic treatment with 200 mL CC-plasma day 59 and 91. The prophylactic administration of CC-plasma was discontinued based on the robust SARS-CoV-2T-cell response detected in peripheral blood collected day 91. On day 113, peripheral blood T-cell phenotypes were restored with activation levels similar to before the onset of COVID-19 (Fig. 3).

## Discussion and conclusion

Our patient had a strong systemic inflammatory reaction and a severe inflammatory response locally in the airways. Previous studies have reported a mild clinical course of COVID-19 in individuals with XLA [1]. The BTK is also expressed by macrophages and other myeloid cells [8]. Pharmacological BTK-inhibition has been reported to attenuate hyperinflammation in COVID-19, and dysfunctional BTK has been suggested an explanation for mild disease in some patients with XLA [9]. However, the lack of BTK in XLA does not affect ex vivo function of monocytes and polymorphonuclear cells, a finding in line with the strong inflammatory response seen in our patient [8]. It can be hypothesized that the absent humoral response protected our patient from the immunopathology driven by SARS-CoV-2 specific antibodies, resulting in multi-organ failure, despite protracted hyperinflammation [8]. Instead, low avidity T-cells may also have contributed to the inflammatory process [10]. After CC-plasma therapy, there was a dramatic shift in the clinical status of the patient, in line with previous reports on XLA-patients with less severe hyperinflammation [4, 5]. Our case adds evidence to the importance of antibodies in the immunological process of SARS-CoV-2 clearance. Studies performed during 2021 have confirmed the benefit of treatment with SARS-CoV-2 mAbs in COVID-19, particularly in seronegative individuals [11, 12]. Passive administration of SARS-CoV-2 specific IgG opsonize viral particles and may thereby improve viral clearance by affecting natural killer cell cytotoxicity and modulating T-cell interactions with antigen presenting cells and thereby resulting in a more efficient anti-viral T-cell response. Negative nasopharyngeal SARS-CoV-2 PCR day 49, and improvement of the T-cell phenotype day 56, with decreasing expression of the checkpoint PD-1, may also reflect effects of the CC-plasma therapy. By 100 days post-infection, the patient’s T cell phenotypes were considered fully recovered, after comparison with samples collected prior to the COVID-19 pandemic. The presence of CMV-virus specific T-cells at this time point, may also indicate functional recovery of the T-cell compartment. CC-plasma therapy is considered safe but does not affect the clinical status in immunocompetent hospitalized patients with COVID-19 [13]. In COVID-19, administration of CC-plasma against SARS-CoV-2 within 72 h after onset of symptoms, reduces severe disease in elderly [14]. Presence of a SARS-CoV-2 specific T-cell response indicates ability to control the infection, still antibody therapy with SARS-CoV-2 specific mAbs, or in low to middle income countries with CC-plasma therapy, should be considered to promote viral clearance in patients with antibody deficiencies [11].

Limitations of this case report include the lack of extended immune phenotyping of T-cells and assessment of SARS-CoV-2 specific T-cell responses prior to day 34. In addition, the presence of viremia was never investigated. Bacteria were isolated in the sputum cultures and a bacterial infection may have contributed to the clinical symptoms. The treatment with broad spectrum antibiotics may also have affected the clinical process. There was no access to test for SARS-CoV-2 neutralizing antibodies in the CC-plasma given. However, only plasma with high anti-spike protein titers was used for treatment. The fact that the patient did not receive any anti-viral drugs or anti-inflammatory treatments can be considered strengths of this case, CC-plasma was the only specific treatment given. Also, the long follow up period as well as the access to T-cell phenotype and chest images, prior to COVID-19 diagnosis, can be included in the strengths.

The patient was overwhelmed by the fast recovery of COVID-19 after the initial CC-plasma transfusions. The patient had a relevant fear of re-infection that was alleviated by regular CC-plasma therapy. Plasma-transfusions continued until we found out about his strong specific T-cell response. It was stressful for the patient when the decision to discontinue the prophylactic CC-plasma therapy was made. However, after careful information that his T-cell response reflected protection against COVID-19 he accepted the decision.

We suggest evaluation of specific SARS-CoV-2T-cell responses in patients with severe COVID-19 and primary or acquired antibody deficiencies (XLA, CVID and patients on B-cell depletion therapy). Albeit clinical studies are needed, the presence of specific T-cell responses indicate protective immunity and may reduce the need for pre-exposure prophylaxis [15] and prophylactic therapy with SARS-CoV-2 mAbs, or CC-plasma, in individuals with antibody deficiencies.

## Supplementary Information

**Additional file 1.** Methods.

## Data Availability

All necessary data are presented as tables and figures in the manuscript. Related information can be made available upon request to the corresponding author.

## Abbreviations

BTK: Bruton’s tyrosine kinase
CC-PLASMA: COVID-19 convalescent plasma
CMV: Cytomegaly virus
CVID: Common variable immunodeficiency
HFNO: High flow nasal oxygen
Ig: Immunoglobulin
mAb: Monoclonal antibody
XLA: X-linked agammaglobulinemia
