# Supplementary material for: Specific T-cell responses for guiding treatment with convalescent plasma in severe COVID-19 and humoral immunodeficiency: a case report
Source: BMC Infect Dis. 2022 Apr 11;22:362. doi: 10.1186/s12879-022-07323-4 (PMC8996199; doi:10.1186/s12879-022-07323-4)
Supplement: Supplementary file 1 — Additional file 1. Methods. [file 12879_2022_7323_MOESM1_ESM.pdf]

Specific T-cell responses for guiding treatment with convalescent plasma in severe COVID-19 and humoral immunodeficiency: a case report

Katarina Nyström, MD, Maria Hjort, PhD, Ramona Fust MNSc, Åsa Nilsdotter Augustinsson MD, PhD, Marie Larsson PhD, Katarina Niward MD, PhD and Sofia Nyström MD, PhD

Supplementary Material

Supplementary Methods

T-cell phenotyping of peripheral blood samples were performed by flow cytometry. Antibodies were added to whole blood and samples were lysed with FACS lysing solution (BD Biosciences). SARS-CoV-2 specific T-cells was detected by flow-cytometric assay for specific cell-mediated immune-response in activated whole blood. In brief, sodium-heparinized whole blood was diluted 1:10 in culture medium (RPMI 1640; Gibco, UK) and incubated for seven days with immunogenic class I peptide overlapping pools of the SARS-CoV-2 spike (S), membrane (M), and nucleocapsid proteins (N), respectively (Miltenyi Biotec, Germany). Stimulation with the mitogen phytohemagglutinin (PHA; Sigma-Aldrich, Germany) was used as positive control and culture medium as negative control. Expanded blast-transformed CD4 and CD8 T-cells were quantified by flow cytometry and the use of Trucount tubes (BD Biosciences). Data were acquired using FACS Canto II (BD Biosciences). Kaluza flow cytometry software version 2.1 (Beckman Coulter) was used for data analysis. All monoclonal antibodies were purchased from BD Biosciences, except PD-1 (eBioscience). Precoated interferon-g ELISpot kit was used according to the manufacture instructions (Mabtech, Sweden).

Convalescent plasma was collected from blood donors with confirmed SARS-CoV-2 infection at least 14 days after resolution of symptoms using standard Swedish donor selection guidelines. The donated plasma was analysed by iFlash 1800 YHLO, confirming the presence of high titre anti-spike IgG antibodies.
